# Maternal Food Restriction during Pregnancy and Lactation Adversely Affect Hepatic Growth and Lipid Metabolism in Three-Week-Old Rat Offspring

**DOI:** 10.3390/ijms17122115

**Published:** 2016-12-15

**Authors:** Sangmi Lee, Young-Ah You, Eun Jin Kwon, Sung-Chul Jung, Inho Jo, Young Ju Kim

**Affiliations:** 1 Department of Obstetrics and Gynecology and Ewha Medical Research Institute, Ewha Womans University Medical School, Seoul 07985, Korea; oolbo32@nate.com (S.L.); yerang02@naver.com (Y.-A.Y.); friendkej1004@hanmail.net (E.J.K.); 2 Department of Molecular Medicine and Ewha Medical Research Institute, Ewha Womans University Medical School, Seoul 07985, Korea; 3 Department of Biochemistry, Ewha Womans University Medical School, Seoul 07985, Korea; jungsc@ewha.ac.kr; 4 Department of Molecular Medicine, Ewha Womans University Medical School, Seoul 07985, Korea; inhojo@ewha.ac.kr

**Keywords:** hepatic growth, lipid metabolism, male offspring, maternal food restriction

## Abstract

Maternal malnutrition influences the early development of foetal adaptive changes for survival. We explored the effects of maternal undernutrition during gestation and lactation on hepatic growth and function. Sprague-Dawley rats were fed a normal or a food-restricted (FR) diet during gestation and/or lactation. We performed analyses of covariance (adjusting for the liver weight/body weight ratio) to compare hepatic growth and lipid metabolism among the offspring. Maternal FR during gestation triggered the development of wide spaces between hepatic cells and increased the expression of mammalian target of rapamycin (mTOR) in three-week-old male offspring compared with controls (both *p* < 0.05). Offspring nursed by FR dams exhibited wider spaces between hepatic cells and a lower liver weight/body weight ratio than control offspring, and increased mTOR expression (*p* < 0.05). Interestingly, the significant decrease in expression of lipogenic-related genes was dependent on carbohydrate-responsive element-binding protein, despite the increased expression of sterol regulatory element-binding protein 1 (SREBP1) (*p* < 0.05). This study demonstrated increased expression of key metabolic regulators (mTOR and SREBP1), alterations in lipid metabolism, and deficits in hepatic growth in the offspring of FR-treated dams.

## 1. Introduction

Maternal nutritional status during gestation and lactation influences foetal adaptive changes for survival and plays a critical role in the increased risk of metabolic and cardiovascular diseases later in life [1,2]. During gestation, maternal undernutrition and consumption of a low-protein low-energy diet typically leads to the development of an intrauterine growth-restricted (IUGR) foetus [3,4,5,6]. Such a foetus exhibits accumulation of abnormal hepatic lipids, hyperglycemia, and hyperinsulinemia. This in turn increases the susceptibility to fatty liver, type 2 diabetes, and obesity during adulthood [3,4,5,6]. Maternal food intake during lactation also affects permanent changes in the nutritional and hormonal statuses of the offspring [7]. Changes in satiety signals by maternal malnutrition may affect energy intake or metabolic rate throughout the offspring’s lifetime and have the potential to induce an overweight state and hepatic steatosis in rat offspring [8,9,10]. Although maternal malnutrition is a major factor adversely affecting foetal and neonatal growth and development, with lifelong consequences, the underlying molecular mechanism remains unclear.

The liver is a central organ for cell growth and metabolism. It integrates incoming signals to control the production of glucose and triglycerides (TGs) for energy sources in other organs, and it functions in fuel storage in adipose depots [11]. Insulin is a hormone that stimulates hepatic glycolysis and lipogenesis, while suppressing gluconeogenesis. The serine/threonine protein kinase AKT and mammalian target of rapamycin (mTOR) play critical roles in transducing cell growth, survival and protein synthesis [12,13]. mTOR acts to integrate growth factors, nutrients and energy signals, and it participates in the regulation of protein synthesis and lipogenesis [14]. Recently, adenosine monophosphate-activated protein kinase alpha (AMPKα) has emerged as a key regulator in energy homeostasis, as it mediates cellular adaptation to environmental or nutritional stress factors and regulates mTOR activation [15]. Lipogenesis is regulated by transcriptional factors such as sterol regulatory element-binding protein 1 (SREBP1) and carbohydrate-responsive element-binding protein (ChREBP) [13], which are responsible for regulating the expression of lipogenic genes such as acetyl-CoA carboxylase alpha (ACCα) and fatty acid synthase (FAS) [16,17]. However, comparative studies of the effects of maternal malnutrition during pregnancy and lactation on the hepatic lipid metabolism of the offspring are required to define the molecular mechanism involved and to develop appropriate preventative strategies.

The present study investigated hepatic growth and lipid profiles in male offspring of rats treated with 50% food restriction (FR) during gestation or lactation. We analysed blood lipid and carbohydrate metabolite profiles in the offspring of dams subjected to FR during gestation or lactation. We evaluated hepatic growth and lipid metabolism in three-week-old offspring by hepatic tissue staining and mRNA and protein expression analyses of hepatic growth-related genes.

## 2. Results

### 2.1. Characteristics of Male Rat Offspring Aged Three Weeks

**Figure 1:**
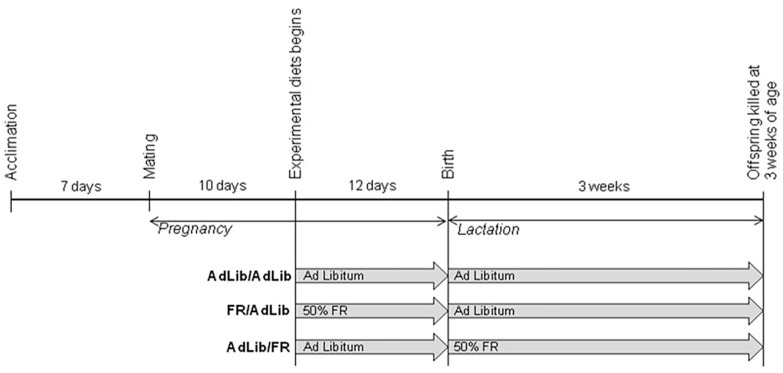
Experimental design. Eight-week-old male and female Sprague-Dawley rats were used in this study. After a one-week acclimation period, the animals were mated. At day 10 of gestation, female rats were divided randomly into three groups: (1) AdLib/AdLib, provided an ad libitum (AdLib, control) diet throughout the experimental period; (2) FR/AdLib, provided a 50% food-restricted diet (FR) during late gestation and an AdLib diet during the lactation period; and (3) AdLib/FR, provided an AdLib diet during late gestation and a FR diet after delivery. After delivery, offspring were weighed and kept together with their respective dams.

On day 10 of gestation, female rats were randomly divided into three groups: AdLib/AdLib control (fed ad libitum throughout the entire experimental period), FR/AdLib (given a 50% FR diet during late gestation and an AdLib diet during lactation), and AdLib/FR (given an AdLib diet during late gestation and an FR diet during lactation) (Figure 1).

Although IUGR offspring had a lower birth weight than controls (*p* < 0.05), three-week-old IUGR offspring nursed by ad libitum (AdLib) dams showed body and liver weights similar to those of the control offspring (Table 1). In contrast, three-week-old offspring nursed by FR dams exhibited a significant decrease (about 33% in body weight and a 20% in liver weight) compared with controls (*p* < 0.05). Three-week-old offspring of AdLib/FR dams exhibited a lower liver-to-body weight ratio than control offspring (*p* < 0.05).

We analysed lipid levels and carbohydrate metabolites in blood from three-week-old offspring to determine the effects of foetal programming. The levels of serum TG, total cholesterol (TC), fasting serum glucose, and insulin were significantly different among the three groups (*p* < 0.05, Table 2). In IUGR offspring nursed by AdLib dams, TG and insulin levels were the highest compared with those from offspring in the other groups (*p* < 0.05). In offspring nursed by FR-fed dams, TC, high-density lipoprotein (HDL)-cholesterol, and low-density lipoprotein (LDL)-cholesterol levels were the highest, while the glucose level was the lowest, among the three groups (*p* < 0.05). After adjusting for the liver weight to body weight ratio, the levels of lipids and glucose differed significantly among the three groups (*p* < 0.05).

### 2.2. Hepatic Growth via the AKT/mTOR Pathway

**Figure 2:**
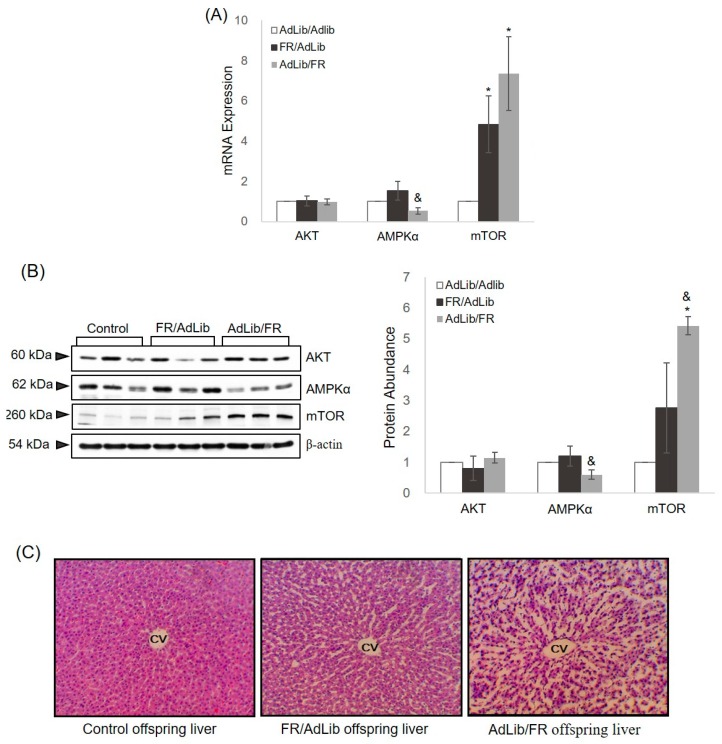
Relative mRNA and protein levels of the hepatic growth-related gene in three-week-old male offspring. (**A**) The liver levels of mRNA encoding AKT, AMPKα, and mTOR were determined by real-time PCR in the three groups (*n* = 9 control, *n* = 9 FR/AdLib, and *n* = 9 AdLib/FR). Each value was calculated using the ΔΔ*C*_t_ method (the cycle threshold value method) and was normalized to that of GAPDH. Data are presented as means ± SDs; (**B**) the protein expression levels of AKT, AMPKα, and mTOR were measured by Western blotting in the three groups (*n* = 9 control, *n* = 9 FR/AdLib, and *n* = 9 AdLib/FR). β-actin served as the reference protein for normalization of band intensity. Data are presented as means ± SDs. *p*-values indicate the significance of the differences among the groups (one-way ANOVA). * (*p* < 0.05) vs. control, and ^&^ (*p* < 0.05) vs. FR/AdLib or AdLib/FR; (**C**) a photomicrograph of offspring livers in the three groups. Liver tissues from male offspring stained with hematoxylin and eosin (H & E) showing dilated and congested central veins. Original magnification, 200×. mTOR, mammalian target of rapamycin; AMPKα, adenosine monophosphate-activated protein kinase alpha; CV, central vein.

We investigated hepatic expression of AKT, AMPKα, and mTOR, and the expression of these genes was normalized to glyceraldehyde-3-phosphate dehydrogenase (GAPDH) expression. In addition, we performed hematoxylin and eosin (H & E) staining to assess hepatic growth and architecture in the three groups. The mRNA and protein expression of AKT in the liver of offspring of FR/AdLib and AdLib/FR dams showed a pattern similar to that of control offspring (Figure 2). The serum insulin level was highest in the FR/AdLib offspring and lowest in the AdLib/FR offspring (Table 2). In the liver from offspring of FR/AdLib dams, both mRNA and protein expression of AMPKα was similar to that in control offspring (Figure 2A,B). However, we observed a significant decrease in hepatic AMPKα expression in offspring of AdLib/FR dams compared with offspring of FR/AdLib dams (*p* < 0.05). Expression of mTOR, the downstream target of AMPKα, was higher in the livers from offspring of FR/AdLib- and AdLib/FR-fed dams than in control offspring (*p* < 0.05). After adjusting for the liver weight to body weight ratio, the expression of hepatic AMPKα and mTOR differed significantly among the three groups (*p* < 0.05, Table S1).

Histopathological analysis of the liver showed a normal architecture in the control offspring, whereas wider spaces between hepatic cells were observed in the FR/AdLib and AdLib/FR offspring. Notably, this structural damage in the liver was more prevalent in the offspring of AdLib/FR-fed dams.

### 2.3. The Regulation of Hepatic Lipogenesis

**Figure 3:**
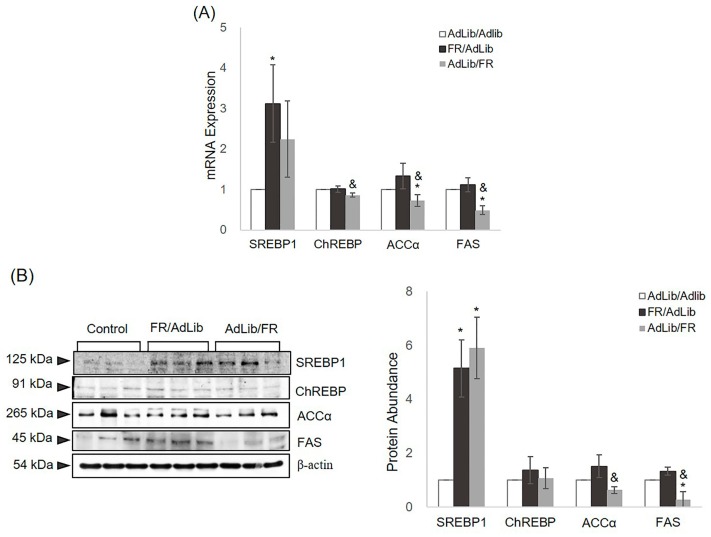
Relative mRNA and protein levels of hepatic lipid metabolism-related genes in three-week-old male offspring. (**A**) The levels of mRNAs encoding SREBP1, ChREBP, ACCα, and FAS were determined by real-time PCR in the three groups (*n* = 9 control, *n* = 9 FR/AdLib, and *n* = 9 AdLib/FR). Each value was calculated using the ΔΔ*C*_t_ method (the cycle threshold value method) normalized to that of GAPDH. Data are presented as means ± SDs; (**B**) the protein expression levels of SREBP1, ChREBP, ACCα, and FAS were measured by Western blotting in the three groups (*n* = 9 control, *n* = 9 FR/AdLib, and *n* = 9 AdLib/FR). β-actin served as the reference protein for normalization of band intensities. Data are presented as means ± SDs. *p-*values indicate the significance of the differences among the groups (one-way ANOVA). * (*p* < 0.05) vs. control, and ^&^ (*p* < 0.05) vs. FR/AdLib or AdLib/FR. SREBP1, sterol regulatory element-binding protein 1; ChREBP, carbohydrate-responsive element-binding protein; ACCα, acetyl-CoA carboxylase alpha; FAS, fatty acid synthase.

We determined the association between serum lipid profiles and hepatic lipogenesis by analysing the mRNA and protein expression levels of SERBP1, ChREBP, ACCα, and FAS, normalized to GAPDH expression. In the liver of offspring of FR/AdLib-fed dams, the mRNA and protein expression levels of SREBP1 were increased due to the presence of increased serum insulin levels (*p* < 0.05, Figure 3). In the offspring of AdLib/FR-fed dams, the protein expression of SREBP1 was also significantly increased compared with that in the control offspring (*p* < 0.05). The mRNA expression of ChREBP was similar between the FR/AdLib and control offspring, whereas the expression was decreased significantly in the offspring of Adlib/FR-fed dams as a result of decreased glucose levels (*p* < 0.05). Moreover, hepatic mRNA and protein expression levels of ACCα and FAS were increased substantially in the offspring of FR/AdLib-fed dams compared with control offspring, whereas the expression levels were significantly decreased in the offspring of AdLib/FR-fed dams (*p* < 0.05, Figure 3). After adjusting for the liver weight to body weight ratio, the expression levels of hepatic SREBP1 and ACCα were significantly different among the three groups (*p* < 0.05, Table S2).

## 3. Discussion

This study showed that dietary manipulation in dams influenced the blood lipid profiles, glucose levels, and expression of genes related to hepatic growth and lipid metabolism in their three-week-old male offspring. We found that maternal food restriction during late gestation and lactation significantly increased hepatic mTOR expression, involved in hepatic growth in the offspring, and altered SREBP1 expression, thereby controlling lipogenesis. These results suggest that maternal FR during late gestation or lactation causes dysfunctional liver signalling and metabolism. Moreover, the altered hepatic growth and lipogenesis during postnatal development predispose the offspring to insulin resistance, diabetes, and non-alcoholic fatty liver diseases later in life.

Epidemiological studies have demonstrated the associations among the early nutritional environment, postnatal growth patterns, and metabolic syndrome in adults [18,19]. Animal models of low birth weight caused by nutrient restriction in the mothers demonstrate an increased risk of adiposity, particularly those who exhibit rapid catch-up growth [20,21]. In our previous studies, we demonstrated catch-up growth in three-week-old rat offspring of FR/AdLib-fed dams, but the offspring of AdLib/FR dams exhibited reduced body and liver weights after the lactation period [21]. Likewise, we found that the serum glucose levels in control offspring were similar to those in the offspring of FR/AdLib-fed dams. However, the significant increase in TG, TC, LDL-cholesterol, and insulin suggested increased body fat, and rapid catch-up growth was exhibited in the offspring of FR/AdLib-fed dams in this study. Insulin is the primary hormone responsible for coordinating the metabolic response to nutrient intake [22]. Following food intake, insulin suppresses hepatic glucose production, which provides brain substrates during fasting and directs ingested nutrients into long-term energy stores [22]. In this study, offspring programmed towards a thrifty phenotype during late gestation might perceive normal glucose levels supplied by lactation as excessive during the postnatal period. Thus, the increased insulin level affected hepatic growth and induced enhanced serum TG and TC, resulting from de novo lipogenesis for hepatic energy storage, in the liver of offspring of FR/AdLib-fed dams.

Maternal undernutrition during lactation significantly decreased serum glucose and TG levels and increased TC, HDL- and LDL-cholesterol levels in the offspring of AdLib/FR-fed dams. Food restriction generally induces lipid catabolism to meet energy requirements and results in high levels of free fatty acids in the bloodstream [23]. We speculate that the offspring of AdLib/FR-fed dams actively break down lipids to supply their energy needs, which leads to higher levels of circulating free fatty acids, resulting in higher serum LDL-cholesterol levels. Additionally, maternal protein restriction was associated with changes in maternal milk nutrients. The levels of amino acids involved in gluconeogenesis decreased and the total fatty acid level rose [24]. Fulfilment of the energy requirements of the offspring by milk affected by poor maternal nutrition may play roles in offspring growth and metabolic programming. Thus, maternal malnutrition during pregnancy and lactation could alter the levels of lipids, fasting glucose, and insulin in offspring. These results suggest that maternal food restriction during pregnancy and lactation can present a health risk during the developmental period and promote cardiovascular and metabolic disease later in life.

The liver is a central organ that regulates energy metabolism and growth [11]. We compared hepatic growth and lipid metabolism among groups to determine how programmed events early in life affect the adverse health of offspring. In mammals, AKT, AMPKα, and mTOR are important regulators of growth in response to insulin and insulin-like growth factor signals [25,26]. Our results found similar expression levels of AKT in the offspring liver among the three groups. However, in the liver of AdLib/FR offspring, the AMPKα expression level was decreased in response to insulin, and the mTOR expression level was increased. One of the most important results of this study was the different phenotypes observed in the liver of three-week-old male offspring according to maternal food intake during late gestation and lactation. The increased serum insulin level and liver weight in FR/AdLib offspring suggest that increased mTOR expression regulates liver growth in the offspring of control dams despite the wide spaces between hepatic cells. However, although offspring of AdLib/FR-fed dams showed the decreased insulin levels in serum and reduced liver weight, and the widest spaces between hepatic cells, mTOR expression was still increased. Many studies have reported that specific foetal and neonatal developmental periods are affected by maternal nutrition status, which causes permanent changes in organs and/or regulatory systems [27,28]. Thus, our results suggest that mTOR expression induced by the diet of the mother during the lactation period is a key factor affecting catch-up growth in IUGR offspring and postnatal growth retardation in the liver. Furthermore, changes in hepatic structure and weight, and an altered serum lipid profile, may be associated with risks of cardiovascular and metabolic diseases later in life.

SREBP1 and ChREBP, master regulators of lipo- and sterolgenic gene transcription, regulate the activation of lipogenic enzymes involved in fatty acid biosynthesis, including ACCα and FAS [29,30]. SREBP1 regulation by insulin and ChREBP regulation by glucose at the transcriptional level are responsible for the biosynthesis of cholesterol, fatty acids, and TGs [31,32]. Additionally, fatty acids synthesize hepatic TG through stearoyl-coenzyme A desaturase 1 activity [33]. In this study, elevated expression levels of hepatic mTOR and SREBP1 might lead to free fatty acid, TG, and cholesterol synthesis in the offspring of FR/AdLib. This is supported by the fact that ACCα and FAS were expressed only moderately, and the serum TG level was increased, in the livers of FR/AdLib offspring compared with control and AdLib/FR offspring. However, FR/AdLib and control offspring displayed similar hepatic ChREPB expression levels under similar glucose levels between the two groups.

The increased serum cholesterol level in AdLib/FR offspring may have affected the elevated expression of hepatic SEEBP1, and ChREPB expression was significantly decreased in response to relatively low glucose levels, suggesting dysregulation of the endogenous sterol response pathway. The expression levels of ACCα and FAS were significantly decreased and were dependent on ChREBP expression. A previous study reported that despite the combined absence of SREBP1c and liver X receptor (LXR), an important regulator of the lipogenic pathway, the ChREBP level was sufficient to maintain ACC expression [32]. Another study showed that in the absence of LXR/SREBP1, FAS expression remained significantly elevated in response to a high carbohydrate diet [33]. Similar to these studies, our results suggested that ChREBP expression in response to the glucose level is more sensitive to the lipogenic pathway than is hepatic SREBP1. However, further studies are required to elucidate why hepatic ACCα and FAS were decreased while SREBP1 was increased in AdLib/FR offspring.

## 4. Materials and Methods

### 4.1. Animals and Study Design

Eight-week-old male and female Sprague-Dawley rats (purchased from Orient Bio Inc., Seongnam, Korea) were mated after a 1-week acclimation period of ad libitum (AdLib) access to unpurified standard laboratory chow (Purina, Pyeongtaek, Korea; Table S3). On day 10 of gestation, female rats were randomly divided into three groups: (1) AdLib/AdLib control (*n* = 3) (fed ad libitum throughout the entire experimental period; (2) FR/AdLib (*n* = 3) (given a 50% FR diet during late gestation and an AdLib diet during lactation); and (3) AdLib/FR (*n* = 3) (given an AdLib diet during late gestation and an FR diet during lactation) (Figure 1). After birth, the offspring remained with their dams. On day 22 of lactation, male offspring were sacrificed by exsanguination under Zoletil anaesthesia (Virbac, Taguig, Philippines) and the livers immediately isolated and stored at −80 °C. Male offspring from control (*n* = 9), FR/AdLib (*n* = 9), and AdLib/FR (*n* = 9) dams were randomly chosen for further analyses. The study was approved by the Animal Research Committee of the School of Medicine at Ewha Womans University (ESM12-0202, 06 September 2012) and was performed in accordance with dictates of the International Guide for the Care and Use of Laboratory Animals.

### 4.2. Analysis of Lipid Profiles in Plasma

Blood samples were collected from 3-week-old male offspring. Nine males from each group were fasted overnight, and blood was collected via cardiac puncture into heparinised tubes for determination of serum TG, TC, HDL- and LDL-cholesterol, glucose, and insulin levels. TG levels were measured using the Pureauto S TG-N kit (Sekisui Medical, Tokyo, Japan), TC levels using the Pureauto S CHO-N kit (Sekisui Medical), HDL-cholesterol levels using the Cholestest N HDL kit (Sekisui Medical), and LDL-cholesterol levels using the LDLC (Sekisui Medical) kit. Glucose concentrations were measured using the Pureauto S Glu (Sekisui Medical) kit on an autoanalyser (HITACHI 7600-210 or HITACHI 7180, Tokyo, Japan). Insulin levels were measured using an insulin kit (Alpco, Salem, NH, USA) according to the manufacturer’s instructions, with the aid of a microplate reader (VersaMax ELISA, Molecular Devices, Sunnyvale, CA, USA). The inter- and intra-assay coefficients of variation for TG, TC, HDL-cholesterol, LDL-cholesterol, and insulin levels were within 5%–10%, corresponding to those specified by the kit manufacturers. Measurements were repeated in 10% of all samples (selected randomly) to confirm reliability.

### 4.3. Quantitative Real-Time Reverse Transcription (RT) Polymerase Chain Reaction (PCR)

Total RNA was isolated from the liver tissues of male offspring (*n* = 9) using the Trizol reagent (Invitrogen, Carlsbad, CA, USA) according to the manufacturer’s instructions. For RT, 1 μg amounts of RNA were converted into cDNA using SuperScript III reverse transcriptase (Invitrogen) and RNasin (Promega, Madison, WI, USA) in 20-μL reaction mixtures. Quantitative PCR was next performed using the PRISM 7000 sequence detection system (Applied BioSystems, Foster City, CA, USA). Each 20-μL reaction mixture contained cDNA, 200 nM of specific primers, and SYBR Premix EX Taq (Takara Bio, Shiga, Japan). PCR proceeded at 95 °C for 10 min; followed by 40 cycles of 95 °C for 15 s, annealing at 62 °C for 1 min, denaturation at 95 °C for 15 s, 62 °C for 20 s, and 95 °C for 15 s. Gene expression levels were calculated using the ΔΔ*C*_t_ method (the cycle threshold value method); the gene encoding GAPDH served as an internal control. The PCR primer pairs are listed in Table S4.

### 4.4. Western Blotting

The livers of 3-week-old male offspring were homogenized in RIPA buffer (Biosesang, Seongnam, Korea) containing a protease inhibitor cocktail (Roche Diagnostics GmbH, Mannheim, Germany), and the supernatants were collected by centrifugation (16,600× *g* for 15 min at 4 °C) (*n* = 9). Protein concentrations were determined using a bicinchoninic acid (BCA) protein assay kit (Thermo Scientific, Rockford, IL, USA). Samples containing 20 μg protein were applied to an 8% (*w*/*v*) sodium dodecylsulphate–polyacrylamide gel. After electrophoresis, the proteins were transferred to nitrocellulose membranes (Whatman, Dassel, Germany) and probed with primary antibodies against AKT, AMPKα, and ACCα (Cell Signalling Technology, Inc., Boston, MA, USA). mTOR was purchased from Merck Millipore (County Cork, Ireland). SREBP1, ChREBP, FAS, and beta-Actin were purchased from Santa Cruz Biotechnology (Santa Cruz, CA, USA). Beta-actin served as the reference protein for normalization of band intensity. All bands were visualized using the SuperSignal West Pico Chemiluminescent Substrate (Thermo Scientific).

### 4.5. Histology

Liver tissues were removed from male offspring on day 22 of lactation, fixed in 4% (*v*/*v*) paraformaldehyde, embedded in paraffin, sectioned into 4-μm-thick slices, placed on glass slides, deparaffinised, and stained with haematoxylin and eosin (H & E). The sections were examined under a light microscope at 200× magnification.

### 4.6. Statistical Analyses

Data were analysed using SPSS ver. 21.0K (SPSS Inc., Chicago, IL, USA) and are expressed as means ± standard deviations (SDs). Differences among the three groups were evaluated by one-way analysis of variance (ANOVA) followed by Tukey’s post-hoc test (*p* < 0.05). Analyses of covariance were adjusted by the ratio of liver to body weight (*p* < 0.05).

## 5. Conclusions

In conclusion, maternal food restriction during late gestation or lactation had different impacts on hepatic growth and lipid metabolism in three-week-old male offspring. These results suggest that rapidly growing foetuses and neonates reared in the presence of suboptimal nutrition during the lactation period exhibit programmed changes in organ structure, cellular responses, and gene expression, which affect the metabolism and physiology of the offspring.

## Figures and Tables

**Table 1:** Comparison of body and liver weights in male offspring of dams fed ad libitum (AdLib) or food restriction (FR) diets during pregnancy and lactation.

Variables	Control (*n* = 9)	FR ^†^/AdLib ^‡^ (*n* = 9)	AdLib ^†^/FR ^‡^ (*n* = 9)
Birth weight (g)	7.33 ± 0.29 ^a^	6.67 ± 0.29 ^b^	7.63 ± 0.32 ^a^
Three-week-old	–	–	–
Body weight (g)	56.97 ± 3.97 ^a^	64.14 ± 4.66 ^a^	18.88 ± 0.21 ^b^
Liver weight (g)	2.61 ± 0.56 ^a^	2.38 ± 0.29 ^a^	0.49 ± 0.02 ^b^
Liver weight to body weight ratio	0.046 ± 0.009 ^a^	0.037 ± 0.003 ^a,b^	0.026 ± 0.001 ^b^

^†^ The maternal diet from day 10 of pregnancy to delivery. ^‡^ The maternal diet during the three-week lactation period. Data are presented as means ± SDs. FR, food restriction, AdLib, ad libitum. ^a,b^ significant differences by one-way analysis of variance following Tukey’s post hoc test (*p* < 0.05).

**Table 2:** Comparison of the plasma lipid and carbohydrate profiles in the offspring of AdLib- or FR-fed dams during pregnancy and lactation.

Variables	Control	FR ^†^/AdLib ^‡^	AdLib ^†^/FR ^‡^	*F*-Value	*F*-Value
	(Mean ± SD)	(Mean ± SD)	(Mean ± SD)	ANOVA	ANCOVA
Lipid profiles	–	–	–	–	–
Triglyceride (mg/dL)	101.3 ± 15.5 ^a^	157.3 ± 16.9 ^b^	50.3 ± 19.5 ^c^	28.42 *	37.26 *
Total cholesterol (mg/dL)	104.0 ± 17.0 ^a^	151.0 ± 9.8 ^b^	444.0 ± 19.0 ^c^	408.95 **	777.19 **
HDL-cholesterol (mg/dL)	36.0 ± 6.0 ^a^	39.7 ± 3.5 ^a^	50.0 ± 1.0 ^b^	9.62 *	25.66 *
LDL-cholesterol (mg/dL)	25.0 ± 3.0 ^a^	48.0 ± 3.6 ^b^	196.0 ± 7.0 ^c^	1091.70 **	1226.96 **
Carbohydrate metabolism	–	–	–	–	–
Glucose (mmol/L)	18.9 ± 0.9 ^a^	21.9 ± 1.9 ^a^	9.8 ± 0.9 ^b^	71.86 **	73.07 **
Insulin (μU/mL)	4.5 ± 1.5 ^a^	10.6 ± 4.3 ^b^	3.1 ± 0.5 ^a^	6.65 *	4.02

^†^ The maternal diet from day 10 of pregnancy to delivery. ^‡^ The maternal diet during the 3-week lactation period. FR, food restriction, AdLib, ad libitum. ^a–c^ significant differences by one-way analysis of variance following Tukey’s post hoc test (*p* < 0.05). Analysis of covariance was adjusted for the liver weight to body weight ratio. * (*p* < 0.05), ** (*p* < 0.001). HDL, high density lipoprotein; LDL, low density lipoprotein.

## Supplementary Materials

Supplementary materials can be found at www.mdpi.com/1422-0067/17/12/2115/s1.
