# Supplementary material for: Maternal Food Restriction during Pregnancy and Lactation Adversely Affect Hepatic Growth and Lipid Metabolism in Three-Week-Old Rat Offspring
Source: Int J Mol Sci. 2016 Dec 15;17(12):2115. doi: 10.3390/ijms17122115 (PMC5187915; doi:10.3390/ijms17122115)
Supplement: Supplementary file 1 [file ijms-17-02115-s001.pdf]

Supplementary Materials: Maternal Food Restriction during Pregnancy and Lactation Adversely Affect Hepatic Growth and Lipid Metabolism in 3-Week-Old Rat Offspring

Sangmi Lee, Young-Ah You, Eun Jin Kwon, Sung-Chul Jung, Inho Jo and Young Ju Kim

Table S1. Comparison of the expressions of hepatic growth related genes.

Variables	mRNA Expression				Protein Expression			
	ANOVA		ANCOVA		ANOVA		ANCOVA	
	F-Value	*p*-Value	*F*-Value	*p*-Value	*F*-Value	*p*-Value	*F*-Value	*p*-Value
AKT	0.068	0.935	0.137	0.875	1.360	0.326	0.697	0.541
AMPKα	8.983	0.016	6.445	0.041	6.513	0.031	4.340	0.081
mTOR	17.138	0.003	8.983	0.022	21.001	0.002	11.087	0.015

Analysis of covariance was adjusted liver weight to body weight ratio.

Table S2. Comparison of the expressions of hepatic lipogenesis related genes.

Variables	mRNA Expression				Protein Expression			
	ANOVA		ANCOVA		ANOVA		ANCOVA	
	F-Value	*p*-Value	*F*-Value	*p*-Value	*F*-Value	*p*-Value	*F*-Value	*p*-Value
SREBP1	5.663	0.042	4.572	0.074	32.390	0.001	20.412	0.004
ChREBP	6.842	0.028	2.809	0.152	0.900	0.455	0.815	0.494
ACCα	9.372	0.014	4.194	0.085	7.567	0.023	4.992	0.064
FAS	9.614	0.013	5.176	0.061	24.210	0.001	12.525	0.011

Analysis of covariance was adjusted liver weight to body weight ratio.

Table S3. Chemical composition of Purina Lab. rodent chow.

Nutrients	Unit	Content	Nutrients	Unit	Content
Protein	%	20.00	Iodine	ppm	1.42
Arginine	%	1.26	Chromium	ppm	0.00
Cystine	%	0.37	Selenium	ppm	0.32
Glycine	%	0.87	–	–	–
Histidine	%	0.50	Vitamins	–	–
Isoleucine	%	0.82	Vitamin K	ppm	6.69
Leucine	%	1.47	Thyamin Hydrichloride	ppm	11.02
Lysine	%	1.01	Riboflavin	ppm	11.57
Methionine	%	0.33	Niacin	ppm	217.70
Phenylalanine	%	0.98	Pantothenic Acid	ppm	88.72
Tyrosine	%	0.63	Choline Chloride	ppm	3447.96
Threonine	%	0.72	Folic Acid	ppm	13.60
Tryptophan	%	0.25	Pyridoxine	ppm	11.00
Valine	%	0.91	Biotin	ppm	0.15
–	–	–	B12	ppm	41.00
Fat (ether extract)	–	4.50	Vitamin A	IU/g	28.03
Linoleic Acid	%	1.10	Vitamin D3 (added)	IU/g	4.00
Linolenic Acid	%	0.12	Vitamin E	IU/kg	100.00
Arachidonic Acid	%	0.02	–	–	–
Omega-3 Fatty Acids	%	1.11	–	–	–
–	–	–	–	–	–

Table S3. *Cont.*

Nutrients	Unit	Content	Nutrients	Unit	Content
Fiber (Crude)	%	6.00	–	–	–
Minerals	–	–	–	–	–
Ash	%	7.25	–	–	–
Calcium	%	1.20	–	–	–
Phosphorus	%	0.62	–	–	–
Phosphorus (non-phytate)	%	0.40	–	–	–
Potassium	%	0.82	Calories Provided by:	–	–
Magnesium	%	0.16	Protein	%	24.52
Sulfur	%	0.22	Fat (ether extract)	%	12.41
Sodium	%	0.34	Carbohydrates	%	63.07
Chloride	%	0.47	–	–	–
Fluorine	ppm	21.38	* Nutrients expressed as percent of ration except where otherwise indicated. Moisture content is assumed to be 10% for the purpose of calculations.		
Iron	ppm	112.93			
Zinc	ppm	128.85			
Manganese	ppm	95.49			
Copper	ppm	22.74			
Cobalt	ppm	0.76			

Table S4. List of primers used in this study.

Genes	Primer Category	Primer Sequence (5′ → 3′)
AKT	Forward	GGT GAT CCT GGT GAA GGA GA
	Reverse	TTG GCG TAC TCC ATG ACA AA
AMPKα	Forward	TGC ACA CAT GAA TGC AAA GA
	Reverse	GAG TTC CAC ACA GCA AAG CA
mTOR	Forward	AGC CGT TGT TGC AGA GAC TT
	Reverse	CAT GGT TCA TGG TGT CTT GC
SREBP1	Forward	GTA CCT GCG GGA CAG ATT AG
	Reverse	TCA GGT CAT GTT GGA AAC AC
ChREBP	Forward	GCA TCC TCA TCC GAC CTT TA
	Reverse	AGA GCA GGC AGG GAA CAG TA
FAS	Forward	TGG CTT CCG TTC AGT CTC TT
	Reverse	CAG TGC CAA GGT CTC TAG CC
ACCα	Forward	AAA GTC CTT GGT CGG GAA GT
	Reverse	TGT GGG AAC AAA CTC GAT GA
